# Maneuverable and Efficient Locomotion of a Myriapod Robot with Variable Body-Axis Flexibility via Instability and Bifurcation

**DOI:** 10.1089/soro.2022.0177

**Published:** 2023-10-16

**Authors:** Shinya Aoi, Yuki Yabuuchi, Daiki Morozumi, Kota Okamoto, Mau Adachi, Kei Senda, Kazuo Tsuchiya

**Affiliations:** ^1^ Department of Mechanical Science and Bioengineering, Graduate School of Engineering Science, Osaka University, Toyonaka, Japan.; ^2^ Department of Aeronautics and Astronautics, Graduate School of Engineering, Kyoto University, Kyoto, Japan.

**Keywords:** myriapod robot, maneuverability, variable stiffness, instability, bifurcation

## Abstract

Legged robots have remarkable terrestrial mobility, but are susceptible to falling and leg malfunction during locomotion. The use of a large number of legs, as in centipedes, can overcome these problems, but it makes the body long and leads to many legs being constrained to contact with the ground to support the long body, which impedes maneuverability. A mechanism for maneuverable locomotion using a large number legs is thus desirable. However, controlling a long body with a large number of legs requires huge computational and energy costs. Inspired by agile locomotion in biological systems, this study proposes a control strategy for maneuverable and efficient locomotion of a myriapod robot based on dynamic instability. Specifically, our previous study made the body axis of a 12-legged robot flexible and showed that changing the body-axis flexibility produces pitchfork bifurcation. The bifurcation not only induces the dynamic instability of a straight walk but also a transition to a curved walk, whose curvature is controllable by the body-axis flexibility. This study incorporated a variable stiffness mechanism into the body axis and developed a simple control strategy based on the bifurcation characteristics. With this strategy, maneuverable and autonomous locomotion was achieved, as demonstrated by multiple robot experiments. Our approach does not directly control the movement of the body axis; instead, it controls body-axis flexibility, which significantly reduces computational and energy costs. This study provides a new design principle for maneuverable and efficient locomotion of myriapod robots.

## Introduction

Animals exhibit agile locomotion using their legs and show remarkable terrestrial mobility for traversing diverse environments. Inspired by animal locomotion, many legged robots have recently been developed to achieve agile locomotion in various environments.^1–7^ In addition, legged robots are expected to be deployed in a wide variety of scenarios, such as search and rescue,^8,9^ hazardous environment operation and exploration,^10,11^ and planetary exploration.^12–14^ However, legged robots are susceptible to falling due to the intermittent repetition of foot contact and foot lift-off,^15,16^ which may result in the breakdown of mechanical and electrical components, from which it is difficult to recover. Furthermore, malfunction even in one leg greatly deteriorates their locomotor performance.^17^ To overcome these problems and achieve high traversability on rough terrain and high tolerance to falling and leg malfunction, greatly increasing the number of legs is useful, as observed in centipedes and millipedes.^18–28^

Although the use of a large number legs has advantages for legged robots, it makes the body long and complicates the interaction with the environment. In particular, many contact legs are physically constrained to remain on the ground to support the long body during locomotion, which impedes maneuverability. Recently, passive components in the body segments and legs have been suggested to contribute to agile myriapod locomotion.^23,26,29^ However, the underlying mechanism of agile locomotion using a large number of legs remains largely unclear from both biological and engineering viewpoints.^30^ Maneuverable locomotion for robots using a large number of legs remains challenging.

In addition to the maneuverability problem, the use of a large number legs greatly increases the number of degrees of freedom of robots, which makes motion planning and control difficult. In particular, conventional controllers precisely plan the motion of all degrees of freedom (e.g., how the long body is bent, where each foot touches the ground, the order in which the legs move) and control the robot to stabilize the desired motion.^31,32^ However, this approach requires huge computational and energy costs. Efficient locomotion for robots using a large number of legs is also challenging.

Recently, bio-inspired approaches, that is, the use of mechanisms elucidated from biological systems, have attracted attention to achieve high locomotor performance of legged robots.^33–38^ Among animals, cockroaches show incredibly agile locomotion using their six legs.^30,39^ It has been suggested that they manipulate the position of ground reaction forces entering the body to control the stability of a straight walk and that straight walk instability helps them to turn quickly.^40,41^ The dynamic instability induces rapid and large movement changes. Inspired by their turning strategy, this study proposes a control scheme for maneuverable and efficient locomotion of myriapod robots. Specifically, we made the body axis of a 12-legged robot flexible. Our previous study^42^ showed that changing the body-axis flexibility produces pitchfork bifurcation. This bifurcation not only induces the dynamic instability of a straight walk but also the transition into a curved walk. These bifurcation characteristics helped the myriapod robot turn quickly. In this study, the robot incorporates a variable stiffness mechanism into the body axis, which allows the robot to change its body-axis flexibility and control the bifurcation characteristics during walking. We developed a simple control strategy based on these properties, which enables the robot to achieve maneuverable and autonomous locomotion. Our approach does not directly control the movement of the body axis; instead, it controls body-axis flexibility, which largely reduces the computational and energy costs. This study provides a new design principle for maneuverable and efficient locomotion of myriapod robots.

## Myriapod Robot

### Robot

**FIG. 1.**
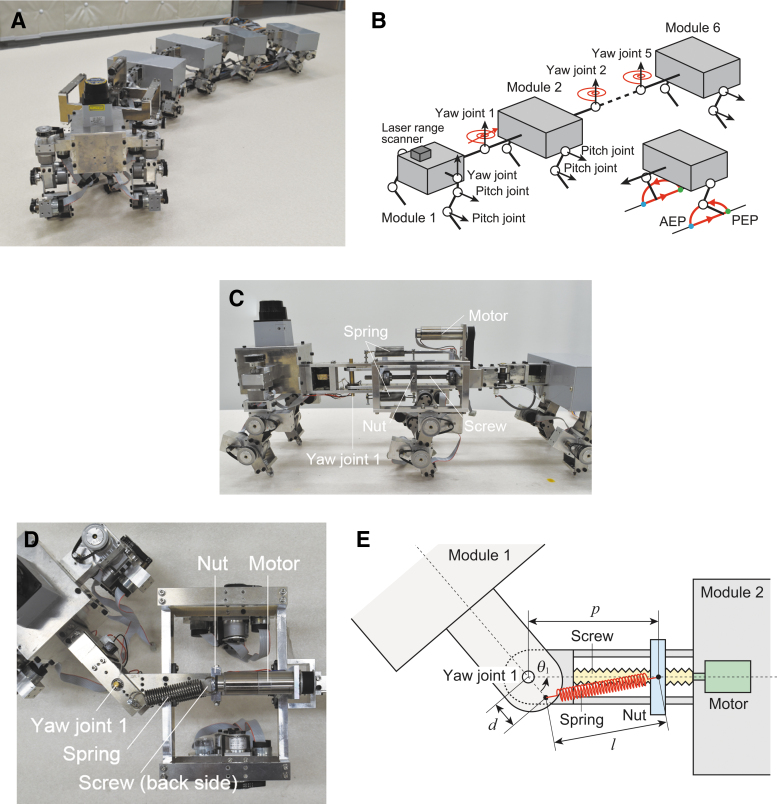
Myriapod robot. **(A)** Photograph and **(B)** schematic model. This robot consists of six modules. Each module has one body segment and one pair of legs. The modules are connected by yaw joints onto which torsional springs are installed. Each leg is controlled by two pitch joints so that the leg tip follows a desired trajectory. A variable stiffness mechanism is incorporated into yaw joint 1. **(C)** Side view, **(D)** top view, and **(E)** schematic model of the variable stiffness mechanism.

We used a multi-legged robot with 6 body segments and 12 legs (Fig. 1A, B). The developed robot^43^ was later improved.^42,44^ In this study, the robot newly incorporated a variable stiffness mechanism. This robot is composed of six modules (modules 1–6). The total mass and length are 9.1 kg and 135 cm, respectively. Each module has a single body segment and one pair of legs. The modules are connected passively through yaw joints (yaw joints 1–5), where torsional springs (spring constant of *k_i_* [i=1,…,5]) and potentiometers are installed. The angles of yaw joints are 0 when the modules are aligned. The gap between the modules is constant. Each leg consists of two links connected by pitch joints. The legs of module 1 have an additional yaw joint for a supplementary control of the walking direction during turning tasks. Each leg joint is controlled by an encoder-equipped motor. Module 1 possesses a laser range scanner (Hokuyo; URG-04LX) to get the relative positions of targets for turning.

The robot walked on a flat wooden floor covered by a vinyl mat to suppress slipping. An external computer (Intel Pentium 4 2.8 GHz, RT-Linux) controlled the robot with 2-ms intervals. The electric power and control signals were provided through cables, which were slack to avoid disturbing the locomotion.

### Variable joint stiffness mechanism

In this study, our robot incorporated a variable stiffness mechanism^45^ in body-segment yaw joint 1 to autonomously change *k*_1_ during robot experiments. The variable stiffness mechanism consists of a ball screw mechanism and a linear tension spring (length: *l*, natural length: $l_{0}=72.5$ mm, spring constant: κ=0.637 N/mm, and initial tension: $f_{0}=10.8$ N), as shown in Figure 1C–E. The ball screw mechanism is attached to the second module (module 2). One side of the spring is attached to the first module (module 1) at a distance of d=25 mm from yaw joint 1 (d<l). The other side is attached to the nut of the ball screw mechanism, whose position *p* (distance from yaw joint 1) is controlled by an encoder-equipped motor (p>d). From the spring tension $f=-\kappa \left(l-l_{0}\right)-f_{0}$ and spring length l=p2+d2−2pd c osθ1, the joint torque $\tau_{1}$ produced by this variable stiffness mechanism is given by

(1) τ1=−κ(p2+d2−2pd c osθ1−l0)+f0p2+d2−2pd c osθ1pdsinθ1

Linearization using the joint angle $\theta_{1}$ around $\theta_{1}=0$ gives

(2) $$\tau_{1}=-\frac{\kappa \left(p-d-l_{0}\right)+f_{0}}{p-d}pd\theta_{1}$$

As a result, the joint stiffness (torsional spring constant) *k*_1_ is approximated by

(3) $$k_{1}=\frac{\kappa \left(p-d-l_{0}\right)+f_{0}}{p-d}pd$$

**FIG. 2.**
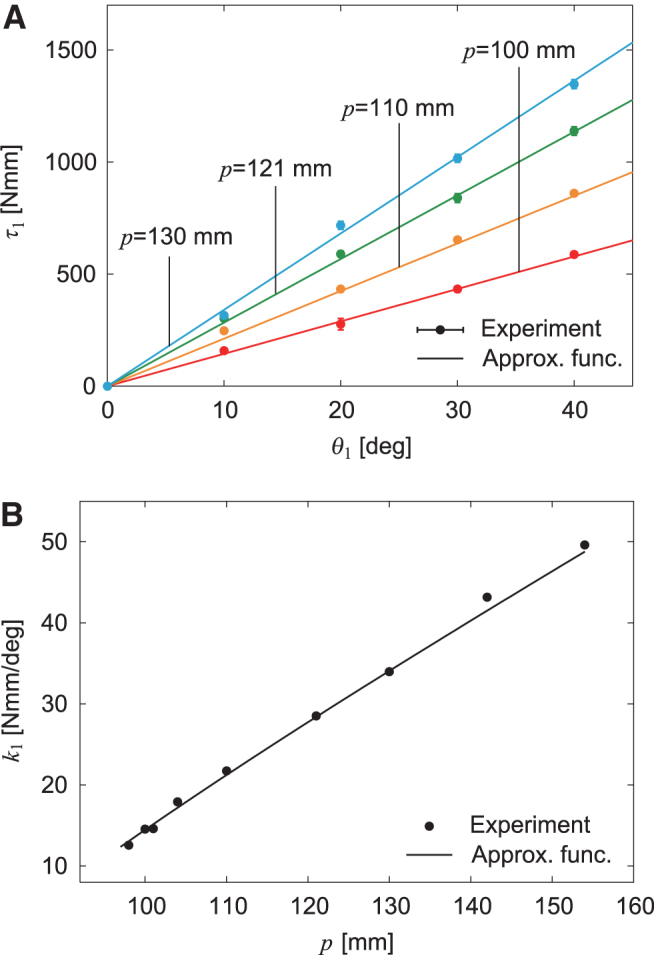
Variable joint stiffness in body-segment yaw joint 1. **(A)** Joint torque $\tau_{1}$ with respect to yaw joint angle $\theta_{1}$ depending on nut position *p*. The data points and error bars are the means and standard deviations, respectively, of five experimental results. **(B)** Spring constant *k*_1_ with respect to *p*. Experimental data were obtained using the least squares method for the $\tau_{1}$−$\theta_{1}$ relationship.

We investigated the properties of the variable stiffness mechanism installed in body-segment yaw joint 1. Specifically, we fixed the position *p* of the nut of the ball screw mechanism at various positions and measured the joint torque $\tau_{1}$ with respect to the joint angle $\theta_{1}$. Figure 2A compares the results with the approximation function.^43^ In addition, we obtained the joint stiffness (torsional spring constant) *k*_1_ from the $\tau_{1}$−$\theta_{1}$ relationship using the least squares method. Figure 2B compares the results with the approximation function.^44^ These experimental results were well fitted by the approximation function. We found that *k*_1_ can be changed from 12 to 50 Nmm/deg by controlling *p* in our system.

### Leg control for straight walking

We used the same controller for the legs as that in our previous study^42,44^ to make the robot walk in a straight line. Specifically, we controlled the two pitch joints of each leg so that the leg tip follows the desired trajectory composed of trajectories for the swing and stance phases (Fig. 1B). The trajectory for the swing phase consists of half of an elliptical curve starting from the posterior extreme position (PEP) and ending at the anterior extreme position (AEP). The trajectory for the stance phase consists of a straight line from the AEP to the PEP, and the leg tips moved at a constant speed parallel to the body segments. We used 0.29 and 0.31 s for the durations of the half elliptical curve and straight line, respectively, and 3 cm for the distance between the AEP and the PEP. We moved the left and right legs in antiphase in each module and used 2π∕3 rad for the relative phase between the ipsilateral legs on adjacent modules. When the yaw joints of the legs in module 1 are fixed so that the leg tips move parallel to the body segments, the robot is expected to walk in a straight line with the body segments parallel to each other because the yaw joints of the body segments have torsional springs and the leg tips move parallel to the body segments at the same speed during the stance phase.

## Instability and Bifurcation of Straight Walking

When large spring constants are used for the body-segment yaw joints, the robot keeps walking in a straight line as expected. Our previous study^43^ revealed that when all the spring constants for the body-segment yaw joints are reduced, the straight walk becomes unstable through Hopf bifurcation (this bifurcation changes the stability of an equilibrium point in a dynamical system by changing a parameter and creating a limit cycle^46^), and body undulations appear, which was verified by a Floquet analysis with a simple robot model. In addition, another previous study^42^ revealed that when only the spring constant *k*_1_ for yaw joint 1 is reduced, the straight walk transitions into a left- or right-curved walk through pitchfork bifurcation (this bifurcation changes the stability of an equilibrium point and creates two equilibrium points^46^), which was also verified by a Floquet analysis with a simple robot model.

In this study, we first confirmed the characteristics of this pitchfork bifurcation in our robot, which was then used for the controllers described in Turning Maneuverability and Autonomous and Maneuverable Locomotion sections below, by comparison with those in a previous study.^42^ Specifically, we used the same spring constant for body-segment yaw joints 2–5 ($k_{i}=41$ Nmm/deg, i=2,…,5) and fixed the spring constant of body-segment yaw joint 1 during the experiments at $k_{1}=15$, 17, 21, 28, or 41 Nmm/deg by a variable stiffness mechanism. All the body segments were set parallel to each other as the initial condition. We fixed the leg yaw joints in module 1 during the experiments.

**FIG. 3.**
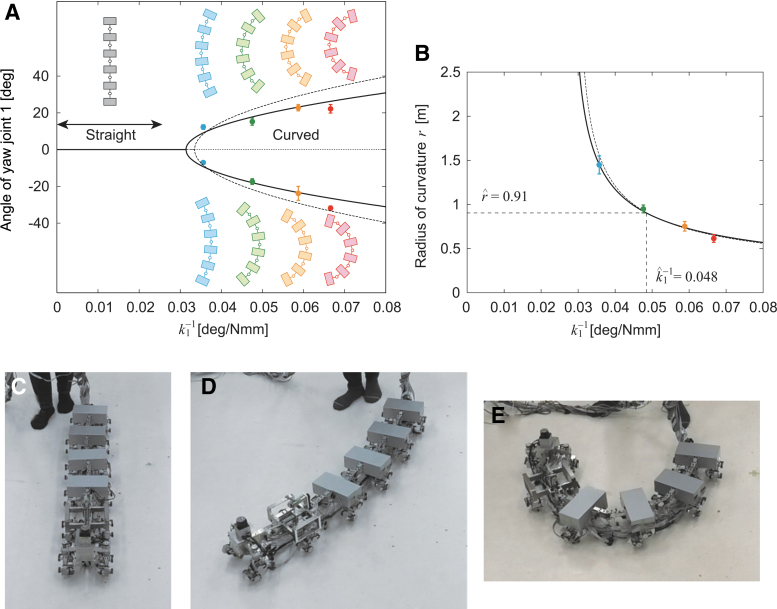
Appearance of curved walk for small *k*_1_ values (Supplementary Movie S1). **(A)** Angle of yaw joint 1 averaged during curved walk for $1∕k_{1}$ that implies pitchfork bifurcation and **(B)** radius of curvature *r* of body axis for $1∕k_{1}$. The data points and error bars are the means and SEs, respectively, of 10 experimental results. *Dotted lines* show the results of a previous study.^42^ Photographs for **(C)** straight walk for $k_{1}>{\hat{k}}_{1}$, **(D)** curved walk with small curvature for $k_{1}∼{\hat{k}}_{1}$, and **(E)** curved walk with large curvature for $k_{1}<{\hat{k}}_{1}$. SE, standard error.

**FIG. 4.**
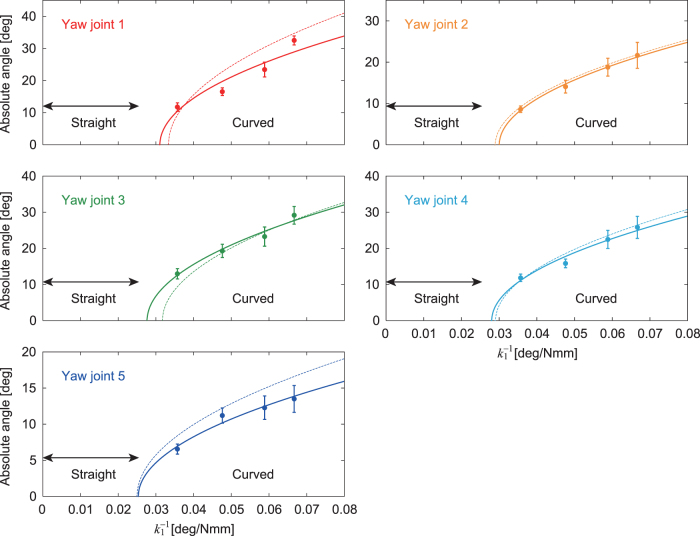
Absolute angles for yaw joints averaged during curved walk for $1∕k_{1}$. The data points and error bars are the means and SEs, respectively, of 10 experimental results. *Dotted lines* show the results of a previous study.^42^

When spring constant *k*_1_ was large ($k_{1}=41$ Nmm/deg), the robot walked in a straight line (Fig. 3A, C, Supplementary Movie S1—Supplementary Appendix SA1 contains links to all movies). However, when *k*_1_ was small ($k_{1}=15$, 17, 21, and 28 Nmm/deg), it walked in a curved line (Fig. 3A, D, E, Supplementary Movie S1) and both left- and right-curved walk were generated. Figure 4 shows the absolute angles of all the body-segment yaw joints for $1∕k_{1}$ averaged over 5 s during the curved walk. These angles increase with $1∕k_{1}$, and all the yaw angles show similar trends. These results suggest that the straight walk becomes unstable and transitions into a curved walk by pitchfork bifurcation. By fitting these angle data with the square root of $1∕k_{1}$, the bifurcation point was estimated to be $k_{1}=35\pm 1.3$ (standard error [SE]) Nmm/deg ($1∕k_{1}=0.028\pm 0.001$ (SE) deg/Nmm) from five body-segment yaw joints. Figure 3B presents the radius of curvature *r* of the body axis during the curved walk for $1∕k_{1}$ determined by $r=5L∕\sum_{i=1}^{5}|\theta_{i}|$, where *L* is the length of each body segment and $\theta_{i}$ is the angle of body-segment yaw joint *i* (i=1,…,5). These bifurcation characteristics are similar to those in a previous study.^42^ These results indicate that we can manipulate the curvature of the body axis for a curved walk by adjusting *k*_1_ by pitchfork bifurcation.

## Turning Maneuverability

### Turning strategy using pitchfork bifurcation

Our previous study^42^ demonstrated that pitchfork bifurcation improves the maneuverability of a myriapod robot during a turning task in which the robot approached one target located on the floor in directions different from those where the robot was oriented. We next confirmed this using our robot and the same one-target task by comparing the results with those in the previous study.^42^

**FIG. 5.**
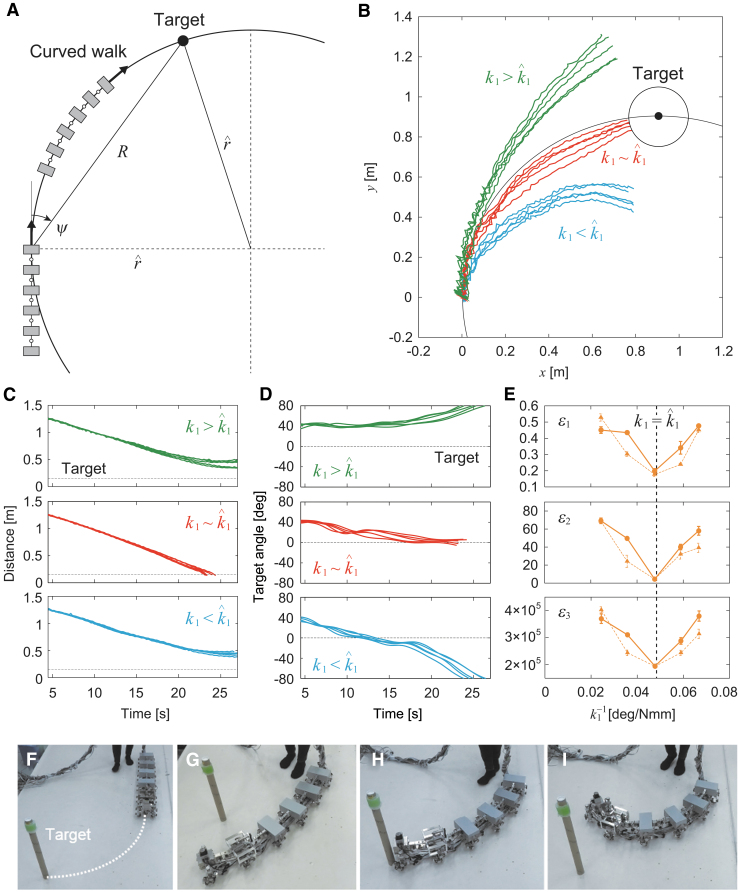
One-target task. **(A)** Schematic model of radius of curvature $\hat{r}$ of curved walk with which the robot approaches a target with relative angle ψ and distance *R*. **(B)** Trajectory of module 1 on the floor, **(C)** target distance, and **(D)** relative target angle of five experimental results for three spring constants with $\psi =45^{\circ }$, R=1.3 m, $\hat{r}=0.91$ m, and $1∕{\hat{k}}_{1}=0.048$ deg/Nmm (Supplementary Movie S2). **(E)** Evaluation criteria $\delta_{1}$, $\delta_{2}$, and $\delta_{3}$ for $1∕k_{1}$. The data points and error bars are the means and SEs, respectively, of five experimental results. *Dotted lines* show the results of a previous study.^42^ Photographs of **(F)** initial condition, **(G)** unsuccessful trial for $k_{1}>{\hat{k}}_{1}$, **(H)** successful trial for $k_{1}∼{\hat{k}}_{1}$, and **(I)** unsuccessful trial for $k_{1}<{\hat{k}}_{1}$.

For any location of a target (relative angle ψ and distance *R*), there is a unique radius of curvature $\hat{r}$ for a curved walk with which the robot will reach the target (Fig. 5A). Since the radius of curvature *r* of the body axis monotonically decreases with $1∕k_{1}$ as shown in Fig. 3B), $k_{1}={\hat{k}}_{1}$ is uniquely determined from $r=\hat{r}$. That is, when ${\hat{k}}_{1}$ is used, the robot spontaneously approaches the target by the pitchfork bifurcation characteristics. This is an optimal turning strategy. However, this strategy is feedforward and depends on the initial conditions for the robot and target. In particular, the initial robot conditions determine the direction in which the robot turns (left or right) due to the pitchfork bifurcation characteristics, which indicates that this strategy does not guarantee the success of the turning task. Therefore, we also used a supplementary turning controller to approach the target using a laser range scanner and leg yaw joints of module 1 developed in our previous study^44^ (Supplementary Appendix SA2). This supplementary controller enabled the robot to approach the targets even when $k_{1}\neq {\hat{k}}_{1}$.

### Turning performance in one-target task

For the initial condition in the one-target task, $\psi =45^{\circ }$ and R=1.3 m were used for the relative angle and distance between module 1 and target, respectively, which generated $\hat{r}=0.91$ m and ${\hat{k}}_{1}=21$ Nmm/deg ($1∕{\hat{k}}_{1}=0.048$ deg/Nmm) (Fig. 5F). The spring constant of yaw joint 1 was fixed during the experiments at $k_{1}=15$, 17, 21, 28, or 41 Nmm/deg by the variable stiffness mechanism and all the body segments were set parallel to each other as the initial condition. Figure 5B shows the trajectory of module 1 on the floor during the turning task for $k_{1}=15$ ($<{\hat{k}}_{1}$), 21 ($∼{\hat{k}}_{1}$), and 41 Nmm/deg ($>{\hat{k}}_{1}$). Figure 5C and D present the time profiles of the target distance and angle relative to the walking direction, respectively. We assumed that the robot reached the target when the distance was <0.15 m and considered this task to be successfully completed. When $k_{1}=41$ Nmm/deg ($>{\hat{k}}_{1}$), it was difficult for the robot to change its walking direction and the trajectory of module 1 bulged outward, so that the robot could not reach the target (Fig. 5G, Supplementary Movie S2). When $k_{1}=15$ Nmm/deg ($<{\hat{k}}_{1}$), the robot quickly changed its walking direction, but moved away from the target because of the small radius of curvature generated by pitchfork bifurcation, and thus could not reach the target (Fig. 5I, Supplementary Movie S2). By contrast, when $k_{1}=21$ Nmm/deg ($∼{\hat{k}}_{1}$), the robot successfully reached the target by the optimal curved walk generated by pitchfork bifurcation (Fig. 5H, Supplementary Movie S2).

To quantitatively clarify the turning performance dependence on *k*_1_, we employed three evaluation criteria, namely $\delta_{1}$, $\delta_{2}$, and $\delta_{3}$, for five spring constants ($k_{1}=15$, 17, 21, 28, and 41 Nmm/deg), as used in our previous study.^42^ Criterion $\delta_{1}$ is the target distance at 23 s (the earliest time when the task is successfully completed), which evaluates how successfully and quickly the robot approached the targets. Criterion $\delta_{2}$ is the absolute value of the target angle relative to the walking direction at 23 s, which evaluates how successfully and quickly the robot was oriented toward the targets. Criterion $\delta_{3}$ is the energy cost of actuators per unit of moving distance and the magnitude of walking direction change, similar to the performance criterion known as the cost of transport, which evaluates the energy efficiency during the task. Specifically, $\delta_{3}=E∕\left(D\Psi \right)$, where *E* is the energy cost calculated using the square of the motor torque as 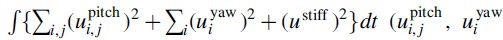, and $u^{stiff}$ are the torques at the leg pitch joints in module *j*, at the leg yaw joints in module 1, and at the variable stiffness mechanism, respectively), *D* is the moving distance calculated by $D_{0}-\delta_{1}$ (*D*_0_ is the target distance at the initial condition), and Ψ is the magnitude of walking direction change calculated by $\Psi_{0}-\delta_{2}$ ($\Psi_{0}$ is the absolute relative target angle at the initial condition). Figure 5E shows the results for $1∕k_{1}$ compared with the results of a previous study.^42^ All criteria show minimum values around $k_{1}={\hat{k}}_{1}$, indicating that the turning strategy using pitchfork bifurcation showed the best performance.

**FIG. 6.**
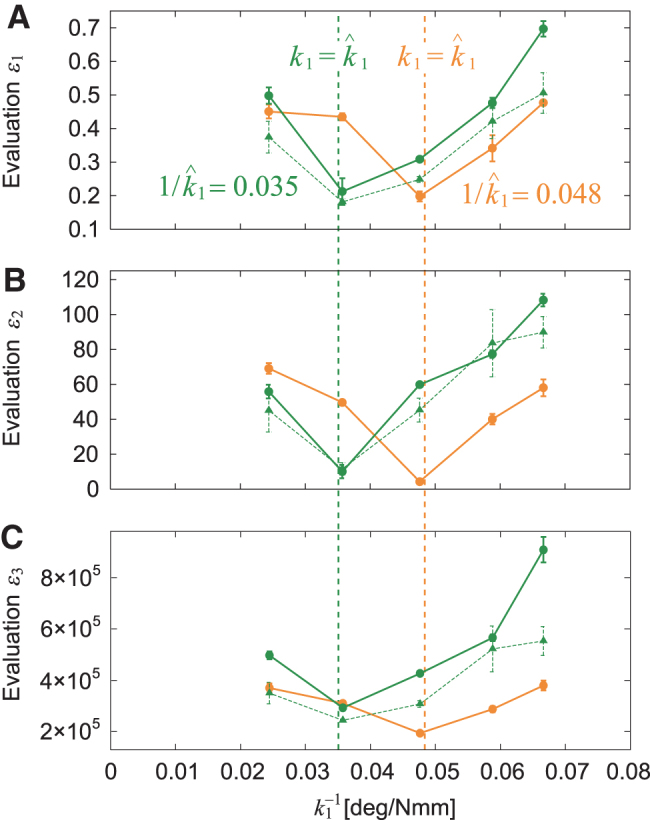
Evaluation criteria **(A)** $\delta_{1}$, **(B)** $\delta_{2}$, and **(C)** $\delta_{3}$ for $1∕k_{1}$ for two different conditions ($1∕{\hat{k}}_{1}=0.035$ and 0.048 deg/Nmm) for one-target task. The data points and error bars are the means and SEs, respectively, of five experimental results. *Dotted lines* show the results of a previous study.^42^

To verify the performance of the turning strategy using pitchfork bifurcation, we also performed the same experiment using different initial conditions for the target, namely $\psi =35^{\circ }$ and R=1.7 m, which yielded $\hat{r}=1.5$ m and ${\hat{k}}_{1}=28$ Nmm/deg ($1∕{\hat{k}}_{1}=0.035$ deg/Nmm). Figure 6A–C compare the evaluation criteria $\delta_{1}$, $\delta_{2}$, and $\delta_{3}$, respectively, for $1∕k_{1}$ with those in a previous study.^42^ All criteria show minimum values around $k_{1}={\hat{k}}_{1}$ and show similar trends to those in Figure 5E.

## Autonomous and Maneuverable Locomotion

### Two-target task for autonomous locomotion

**FIG. 7.**
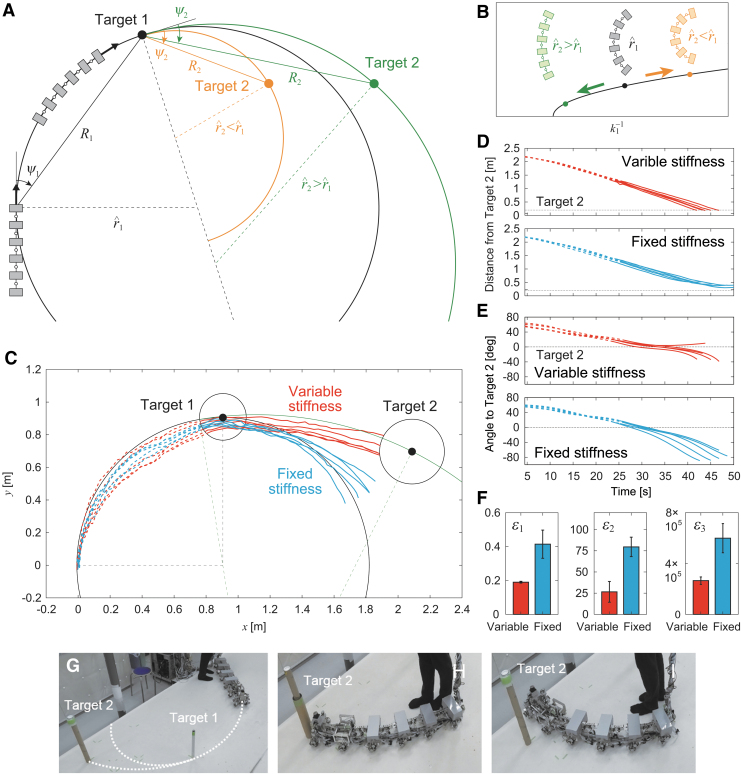
Two-target task with the same direction for targets. **(A)** Schematic model of radius of curvature ${\hat{r}}_{1}$ for target 1 with relative angle $\psi_{1}$ and distance *R*_1_ and radius of curvature ${\hat{r}}_{2}$ for target 2 with relative angle $\psi_{2}$ and distance *R*_2_. **(B)** Change of *k*_1_ depending on ${\hat{r}}_{2}$. **(C**–**I**) Experimental results for larger radius of curvature in target 2 with $\psi_{1}=45^{\circ }$, $R_{1}=1.3$ m, ${\hat{r}}_{1}=0.91$ m, $1∕{\hat{k}}_{11}=0.048$ deg/Nmm, $\psi_{2}=18^{\circ }$, $R_{2}=1.3$ m, ${\hat{r}}_{2}=2.2$ m, and $1∕{\hat{k}}_{12}=0.031$ deg/Nmm (${\hat{r}}_{2}>{\hat{r}}_{1}$) (Supplementary Movie S3). (**C**) Trajectory of module 1 on the floor, **(D)** distance from target 2, and **(E)** relative angle of target 2 of five experimental results for variable and fixed stiffnesses. The *dotted and solid lines* indicate data before and after the robot reached target 1, respectively. **(F)** Evaluation criteria $\delta_{1}$, $\delta_{2}$, and $\delta_{3}$. The data points and error bars are the means and standard deviations, respectively, of five experimental results. Photographs for **(G)** initial condition, **(H)** successful trial with changing stiffness, and **(I)** unsuccessful trial without changing stiffness.

Autonomous locomotion is required for legged robots to allow them to change their destination in accordance with the situation and then produce adequate behavior depending on the destination. We next tackled the autonomous locomotion capability of our robot. To examine whether our robot achieves autonomous locomotion and to evaluate its maneuverability during autonomous locomotion, we used two targets (targets 1 and 2) placed at different locations on the floor (Fig. 7A). The robot first approached the first target (target 1). After the robot had reached target 1 (i.e., the target distance was <0.15 m), the robot approached the second target (target 2). When the distance to target 2 was <0.2 m, the task was considered to be successfully completed.

Although the optimal joint stiffness ${\hat{k}}_{1}$ was uniquely determined in the one-target tasks above, it is not necessarily the same between the two sequential approaches in the two-target tasks. Furthermore, the target direction (left or right) is not necessarily the same between the two approaches. As shown in Figure 3A, after the straight walk becomes unstable due to a change in *k*_1_, it transitions into a left- or right-curved walk by pitchfork bifurcation. Once a curved walk appears, the curve direction does not change unless large external forces are applied. Therefore, the situation in two-target tasks depends on the target direction.

### Two-target task with the same target direction

In this study, we solved the problems related to optimal stiffnesses and target direction by controlling the joint stiffness of body-segment yaw joint 1, that is, we used a variable stiffness mechanism during locomotion. We began with the same target direction for the two approaches. At the beginning of the first approach to target 1, the robot determined the optimal stiffness as ${\hat{k}}_{11}$ by calculating ${\hat{r}}_{1}$ from the relative angle $\psi_{1}$ and the distance *R*_1_ of target 1 (Fig. 7A). After the robot reached target 1, it determined the optimal stiffness as ${\hat{k}}_{12}$ by calculating ${\hat{r}}_{2}$ based on the relative angle $\psi_{2}$ and distance *R*_2_ to target 2 (Fig. 7A) and changed *k*_1_ from ${\hat{k}}_{11}$ to ${\hat{k}}_{12}$ by the variable stiffness mechanism. The joint stiffness increased when ${\hat{r}}_{2}>{\hat{r}}_{1}$ and decreased when ${\hat{r}}_{2}<{\hat{r}}_{1}$ (Fig. 7B).

To examine the performance of the joint stiffness control, we used $\psi_{1}=45^{\circ }$ and $R_{1}=1.3$ m, which yielded ${\hat{r}}_{1}=0.91$ m and ${\hat{k}}_{11}=21$ Nmm/deg ($1∕{\hat{k}}_{11}=0.048$ deg/Nmm), and $\psi_{2}=18^{\circ }$ and $R_{2}=1.3$ m, which yielded ${\hat{r}}_{2}=2.2$ m and ${\hat{k}}_{12}=32$ Nmm/deg ($1∕{\hat{k}}_{12}=0.031$ deg/Nmm) (Fig. 7G). For this condition, ${\hat{r}}_{2}>{\hat{r}}_{1}$. We compared cases with and without a change in *k*_1_ after the robot reached target 1. Figure 7C shows the trajectory of the first module on the floor for these two cases. Figures 7D and E show the time profiles for the distance from target 2 and the relative angle of target 2 with respect to the walking direction, respectively. Without a change in joint stiffness, the curved walk of the robot when approaching target 2 was almost the same as that when approaching target 1, and thus the robot could not reach target 2 (Fig. 7I, Supplementary Movie S3). In contrast, when the robot changed the joint stiffness to the second optimal value, it reached target 2 through sequential optimal curved walks generated by pitchfork bifurcation (Fig. 7H, Supplementary Movie S3).

To quantitatively clarify the difference in performance between the cases with and without a change in joint stiffness, we also compared three evaluation criteria, namely $\delta_{1}$, $\delta_{2}$, and $\delta_{3}$ (Fig. 7F), as used in the one-target tasks. Specifically, $\delta_{1}$ and $\delta_{2}$ were evaluated at 22 s after the robot reached target 1, and $\delta_{3}$ was calculated after the robot reached target 1. Furthermore, we examined whether changing the joint stiffness during the task results in better turning maneuverability than that obtained without changing it using these three criteria and a one-sided *t*-test with Bonferroni correction. The results indicated that all criteria showed smaller values when the joint stiffness was changed. The *p*-values for a one-sided *t*-test were calculated as p=0.002 for $\delta_{1}$, p=0.000 for $\delta_{2}$, and p=0.002 for $\delta_{3}$ (Bonferroni correction α=0.05∕2=0.025), which suggests that changing the joint stiffness leads to better turning maneuverability.

**FIG. 8.**
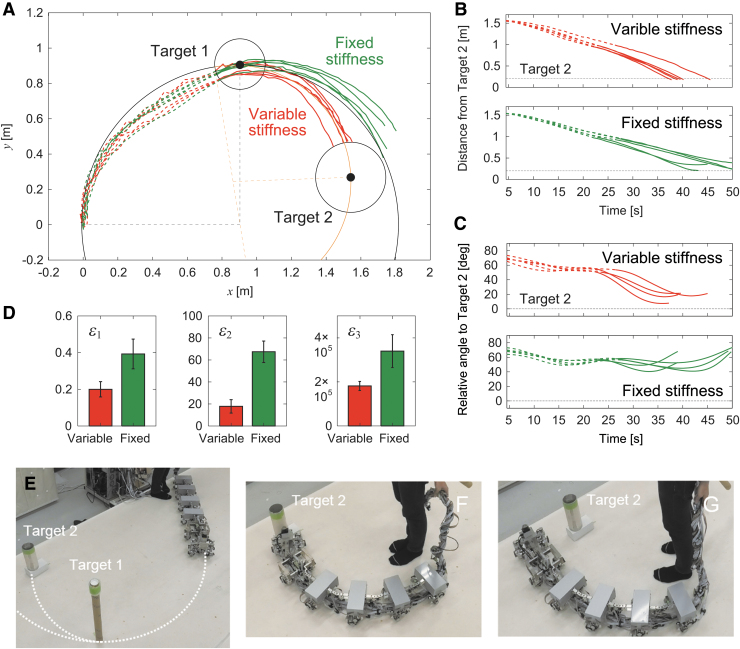
Experimental results for two-target task with the same direction and smaller radius of curvature for target 2 with $\psi_{1}=45^{\circ }$, $R_{1}=1.3$ m, ${\hat{r}}_{1}=0.91$ m, $1∕{\hat{k}}_{11}=0.048$ deg/Nmm, $\psi_{2}=48^{\circ }$, $R_{2}=1.0$ m, ${\hat{r}}_{2}=0.67$ m, and $1∕{\hat{k}}_{12}=0.068$ deg/Nmm (${\hat{r}}_{2}<{\hat{r}}_{1}$) (Supplementary Movie S4). **(A)** Trajectory of module 1 on the floor, **(B)** distance from target 2, and **(C)** relative angle of target 2 of five experimental results for variable and fixed stiffnesses. The *dotted and solid lines* indicate data before and after the robot reached target 1, respectively. **(D)** Evaluation criteria $\delta_{1}$, $\delta_{2}$, and $\delta_{3}$. The data points and error bars are the means and standard deviations, respectively, of five experimental results. Photographs of **(E)** initial condition, **(F)** successful trial with changing stiffness, and **(G)** unsuccessful trial without changing stiffness.

We also performed the same experiment using different conditions for target 2, namely $\psi_{2}=48^{\circ }$ and $R_{2}=1.0$ m, which yielded ${\hat{r}}_{2}=0.67$ m and ${\hat{k}}_{12}=$15 Nmm/deg ($1∕{\hat{k}}_{12}=0.065$ deg/Nmm). For this condition, ${\hat{r}}_{2}<{\hat{r}}_{1}$. We evaluated $\delta_{1}$ and $\delta_{2}$ 16 s after the robot reached target 1 and calculated $\delta_{3}$ after the robot reached target 2. The results show similar trends (Fig. 8, Supplementary Movie S4), which verifies the performance of the proposed controller using the variable stiffness mechanism.

### Two-target task with different target directions

**FIG. 9.**
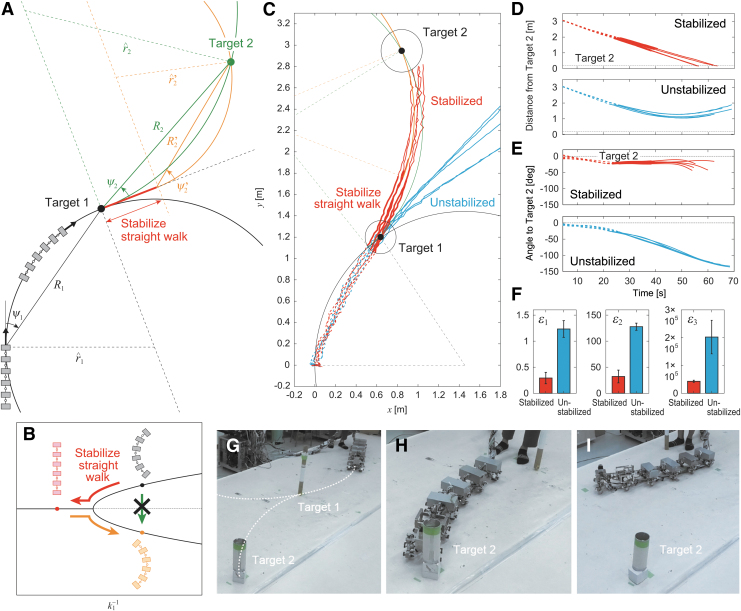
Two-target task with different target directions. **(A)** Schematic model of radius of curvature ${\hat{r}}_{1}$ for target 1 with relative angle $\psi_{1}$ and distance *R*_1_, radius of curvature ${\hat{r}}_{2}$ for target 2 with relative angle $\psi_{2}$ and distance *R*_2_, and radius of curvature $\hat{r}\prime_{2}$ for target 2 with relative angle $\psi \prime_{2}$ and distance $R\prime_{2}$ when stabilizing the straight walk after the robot reaches target 1. **(B)** Two changes of *k*_1_. The straight walk is stabilized once to change the curve direction.“×” indicates that the curve direction cannot change in this route. **(C**–**I**) Experimental results with $\psi_{1}=28^{\circ }$, $R_{1}=1.4$ m, ${\hat{r}}_{1}=1.5$ m, $1∕{\hat{k}}_{11}=0.036$ deg/Nmm, $\psi_{2}=-40^{\circ }$, $R_{2}=1.9$ m, ${\hat{r}}_{2}=1.5$ m, $1∕{\hat{k}}_{12}=0.036$ deg/Nmm, $\psi \prime_{2}=-22\pm 2.1$ (SE)${}^{\circ }$, $R_{2}=1.2\pm 0.02$ (SE) m, ${\hat{r}}_{2}=1.6\pm 0.17$ (SE) m, and $1∕{\hat{k}}_{12}=0.035\pm 0.0012$ (SE) deg/Nmm (Supplementary Movie S5). (**C**) Trajectory of module 1 on the floor, **(D)** distance from target 2, and **(E)** relative angle of target 2 of five experimental results for stabilized and unstabilized straight walks. The *dotted and solid lines* indicate data before and after the robot reached target 1, respectively. Bold lines indicate stabilization of the straight walk. **(F)** Evaluation criteria $\delta_{1}$, $\delta_{2}$, and $\delta_{3}$. The data points and error bars are the means and standard deviations, respectively, of five experimental results. Photographs of **(G)** initial condition, **(H)** successful trial with stabilizing the straight walk, and **(I)** unsuccessful trial without stabilizing the straight walk.

Next, we consider different target directions for the two approaches. After the robot reached target 1, it temporarily increased its joint stiffness by the variable stiffness mechanism to stabilize the straight walk (Figs. 9A, B). The robot then determined the optimal stiffness as $\hat{k}\prime_{12}$ by calculating $\hat{r}\prime_{2}$ based on the relative angle $\psi \prime_{2}$ and distance $R\prime_{2}$ to target 2, and changed *k*_1_ to $\hat{k}\prime_{12}$ by the variable stiffness mechanism (Fig. 9A). These two changes in joint stiffness allowed the robot to change the curve direction (Fig. 9B). As the duration for the temporal stabilization of the straight walk increases, the robot can change the curve direction. However, this changes the relative target position and limits the reachable space. That is, the success rate and reachable space of the task are a trade-off. We determined 14 s for the temporal stabilization of the straight walk through robot experiments so that the robot can change the left-right direction for various conditions.

To examine the performance of this joint stiffness control, we used $\psi_{1}=28^{\circ }$ and $R_{1}=1.4$ m, which yielded ${\hat{r}}_{1}=1.5$ m and ${\hat{k}}_{11}=28$ Nmm/deg ($1∕{\hat{k}}_{11}=0.036$ deg/Nmm), and $\psi_{2}=-40^{\circ }$ and $R_{2}=1.9$ m, which yielded ${\hat{r}}_{2}=1.5$ m and ${\hat{k}}_{12}=$28 Nmm/deg ($1∕{\hat{k}}_{12}=0.036$ deg/Nmm) (Fig. 9G). For this condition, ${\hat{k}}_{11}={\hat{k}}_{12}$. We compared cases with and without stabilizing the straight walk after the robot reached target 1. Figure 9C shows the trajectory of the first module on the floor for the two cases. Figures 9D and E show the time profiles for the distance from target 2 and the angle of target 2 relative to the walking direction, respectively. Without stabilizing the straight walk, the robot could not change the curve direction after it reached target 1 and thus could not reach target 2 (Fig. 9I, Supplementary Movie S5). In contrast, when the robot temporarily stabilized the straight walk and then determined the second optimal stiffness, it reached target 2 through the optimal curved walk generated by pitchfork bifurcation (Fig. 9H, Supplementary Movie S5). The robot used $\psi \prime_{2}=-22\pm 2.1$ (SE)${}^{\circ }$ and $R\prime_{2}=1.2\pm 0.02$ (SE) m, which yielded $\hat{r}\prime_{2}=1.6\pm 0.17$ (SE) m and $\hat{k}\prime_{12}=29\pm 0.97$ (SE) Nmm/deg ($1∕\hat{k}\prime_{12}=0.035\pm 0.0012$ (SE) deg/Nmm).

To quantitatively clarify the difference in performance between cases with and without stabilizing the straight walk, we compared three evaluation criteria, namely $\delta_{1}$, $\delta_{2}$, and $\delta_{3}$ (Fig. 9F). Specifically, $\delta_{1}$ and $\delta_{2}$ were evaluated 31 s after the robot reached target 1, and $\delta_{3}$ was calculated after the robot reached target 2. Furthermore, we examined whether stabilizing the straight walk for different target directions results in better turning maneuverability than that obtained without stabilizing it using these two criteria and a one-sided *t*-test with Bonferroni correction. The results indicated that all criteria showed smaller values when the straight walk was stabilized. The *p*-values for a one-sided *t*-test were calculated as p=0.000 for $\delta_{1}$, p=0.000 for $\delta_{2}$, and p=0.002 for $\delta_{3}$ (Bonferroni correction α=0.05∕2=0.025), which suggest that temporarily stabilizing the straight walk leads to better turning maneuverability.

### Autonomous locomotion

Although these experiments used at most two targets, the results suggest that the proposed method allows the robot to achieve maneuverable locomotion even for multiple sequential targets. That is, the robot achieves autonomous and maneuverable locomotion. To demonstrate this, we performed an experiment where the robot approached nine targets placed on the floor sequentially (Supplementary Movie S6).

## Discussion and Conclusion

Maneuverability and efficiency are critical issues for legged robots. this study focused on dynamic instability to address these issues, inspired by the agile locomotion of cockroaches.^40,41^ Maneuverability is related to the ability to change movement direction. When the movement direction is destabilized during locomotion, the instability provides driving forces to rapidly change the movement direction and thus improves maneuverability. In addition to cockroaches, many animals are thought to use dynamic instability to enhance maneuverability in their locomotion. In particular, because the instability is determined by the body dynamics through interaction with the environment, it is outstanding in locomotion generated through aerodynamics and hydrodynamics, such as the locomotion of flying insects^47–49^ and sea animals.^50–52^ In addition to such biological systems, some fighter aircraft, such as the F-16, are designed to be aerodynamically unstable to increase maneuverability.^53,54^ The use of dynamic instability is thus useful from both biological and engineering viewpoints.

In addition to dynamic instability, this study used pitchfork bifurcation. General myriapod robots use actuators for controlling not only the leg joints but also body-segment joints and calculate the desired motion for all joints.^31,32^ However, this leads to huge computational and energy costs. In contrast, this study developed a myriapod robot, whose body-segment joints are passive because they use torsional springs rather than actuators. Pitchfork bifurcation generated a curved walk, whose curvature is controllable by the body-segment joint stiffness (Figs. 2 and 3). Because the generated curved walk was robust and the gait patterns quickly changed, the bifurcation characteristics greatly enhanced maneuverability (Figs. 5–8). Furthermore, manipulating the stability based on pitchfork bifurcation allowed the robot to change the turning direction (Fig. 9) and perform autonomous locomotion. Although the change in the spring constant of other yaw joints than yaw joint 1 also produces pitchfork bifurcation, we changed that of yaw joint 1 because it was the most effective in the experiments. Our approach does not directly control the movement of the body axis; instead, it controls the body-axis flexibility. These characteristics greatly reduce the computational and energy costs. Dynamic instability and pitchfork bifurcation are characteristics of the dynamical system embedded in our robot. The generation of robot movements by inherent dynamics rather than actuators is crucial for efficient locomotion.^55,56^ Our findings will provide a new design principle for maneuverable and efficient locomotion of myriapod robots. To further clarify the advantages and limitations of the proposed approach, in future studies, we would like to quantitatively investigate how robust the curved walk is, how long it takes to change the gait pattern, and what target arrangements are reachable.

Our robot mainly consists of standard metal and DC motors and is basically hard. Only the torsional springs in the body-segment yaw joints have soft characteristics. However, these soft elements governed the entire dynamics of the robot and created various types of locomotion, which greatly improved the performance. This seems to be a benefit seen in soft robots. We investigated the contribution of the proposed controller to maneuverable and efficient locomotion of a myriapod robot through robot experiments on a hard flat floor. In the future, it will be important to validate our design in more complex environments, such as rough terrain. In particular, although our robot has torsional springs in the body-segment yaw joints, more compliant components should be also incorporated into the body-segment pitch and roll joints and legs, as previously reported.^26,27^

## Supplementary Material

Supplemental data

Supplemental data

Supplemental data

Supplemental data

Supplemental data

Supplemental data

Supplemental data

Supplemental data

## Supplementary Material

Supplementary Appendix SA1

Supplementary Appendix SA2
