# Supplementary material for: Maneuverable and Efficient Locomotion of a Myriapod Robot with Variable Body-Axis Flexibility via Instability and Bifurcation
Source: Soft Robot. 2023 Oct 16;10(5):1028–40. doi: 10.1089/soro.2022.0177 (PMC10616954; doi:10.1089/soro.2022.0177)
Supplement: Supplemental data [file Supp_AppS1.pdf]

Appendix 1. Supplementary movies

We recorded 6 supplementary movies to show the pitchfork bifurcation of a straight walk, turning performance, and autonomous and maneuverable locomotion in the robot experiments:

Movie 1 Straight walk, curved walk with a small curvature, and curved walk with a large curvature using a large spring constant, small spring constant, and very small spring constant, respectively, for the torsional spring in yaw joint 1.

Movie 2 Unsuccessful approaches in a one-target task performed when using a spring constant larger and smaller than the optimal value for yaw joint 1, and successful approach using a spring constant close to the optimal value.

Movie 3 Unsuccessful approach in a two-target task with the same direction and a larger radius of curvature for target 2 performed using a fixed joint stiffness in yaw joint 1, and successful approach using a variable joint stiffness in yaw joint 1.

Movie 4 Unsuccessful approach in a two-target task with the same direction and a smaller radius of curvature for target 2 performed using a fixed joint stiffness in yaw joint 1, and successful approach using a variable joint stiffness in yaw joint 1.

Movie 5 Unsuccessful approach in a two-target task with different directions for target 2 performed without stabilizing the straight walk after reaching target 1, and successful approach by stabilizing the straight walk for a while using a variable joint stiffness in yaw joint 1.

Movie 6 Autonomous and maneuverable locomotion when approaching multiple targets placed on the floor sequentially.
