# Supplementary material for: Maneuverable and Efficient Locomotion of a Myriapod Robot with Variable Body-Axis Flexibility via Instability and Bifurcation
Source: Soft Robot. 2023 Oct 16;10(5):1028–40. doi: 10.1089/soro.2022.0177 (PMC10616954; doi:10.1089/soro.2022.0177)
Supplement: Supplemental data [file Supp_AppS2.pdf]

Appendix 2. Supplementary turning control by leg yaw joints

The optimal turning strategies are feedforward and depend on the relationship between the robot and targets at the onset of approach. In particular, the initial robot conditions determine the direction in which the robot turns (left or right) due to the properties of pitchfork bifurcation, as shown in Fig. 3**A**. To guarantee a successful approach to the targets, we used a supplementary feedback-based controller, which was developed in our previous study[1]. Specifically, we used the laser range scanner to measure the relative target angle ψ of module 1 and the yaw joints of the left and right legs in module 1, whose angles are ψ_1 and ψ_2, respectively. We designed the desired angles $\hat{\psi}_1$ and $\hat{\psi}_2$ for ψ_1 and ψ_2, respectively, for each gait cycle [$t_i^n \leq t < t_i^n + T$ ($i = 1$ and 2 are used for the left and right legs, respectively), where $t = t_i^n$ is the time when the desired leg tip reaches the PEP at the nth gait cycle, and T is the gait cycle duration ($= 0.6$ s)] as follows:

$$\hat{\psi}_i(t) = \begin{cases} \hat{\psi}_i(t_i^n) & t_i^n \leq t < t_i^n + t_{\text{start}} \\ \hat{\psi}_i(t_i^n) + \Delta_i^n \dfrac{t - t_i^n - t_{\text{start}}}{t_{\text{end}} - t_{\text{start}}} & t_i^n + t_{\text{start}} \leq t \leq t_i^n + t_{\text{end}} \\ \hat{\psi}_i(t_i^n + t_{\text{end}}) & t_i^n + t_{\text{end}} < t < t_i^n + T \end{cases}$$

$$\Delta_i^n = \begin{cases} \psi(t_i^n + t_{\text{start}}) - \hat{\psi}_i(t_i^n + t_{\text{start}}) & |\psi(t_i^n + t_{\text{start}}) - \hat{\psi}_i(t_i^n + t_{\text{start}})| < 5^\circ \\ 5^\circ & \psi(t_i^n + t_{\text{start}}) - \hat{\psi}_i(t_i^n + t_{\text{start}}) > 5^\circ \\ -5^\circ & \psi(t_i^n + t_{\text{start}}) - \hat{\psi}_i(t_i^n + t_{\text{start}}) < -5^\circ \end{cases}$$

We used 40% and 80% of the duration of the half elliptical curve ($= 0.12$ and 0.23 s) for t_{start} and t_{end}, respectively. We changed the yaw angle by Δ_i^n toward the target during the swing phase and limited the maximum angle to 5° for one gait cycle. We also limited the maximum angle of the yaw joint to 5° during the turning tasks. This supplementary controller did not intend to force the robot to follow the optimal curved path generated by the turning strategy using pitchfork bifurcation, but intended to modulate the walking direction of module 1 based on the target direction. This solves the issues related to the feedforward properties of the optimal turning strategies and the turning direction determined by the initial robot conditions, and in addition enables the robot to approach the target even for $k_1 \neq \hat{k}_1$.

References

1. Aoi, S., Tanaka, T., Fujiki, S., Funato, T., Senda, K., and Tsuchiya, K. Advantage of straight walk instability in turning maneuver of multilegged locomotion: a robotics approach. *Sci. Rep.*, 6:30199 (2016).
